# Biochemical and biophysical characterization of cell-free synthesized Rift Valley fever virus nucleoprotein capsids enables in vitro screening to identify novel antivirals

**DOI:** 10.1186/s13062-016-0126-5

**Published:** 2016-05-14

**Authors:** Sean Broce, Lisa Hensley, Tomoharu Sato, Joshua Lehrer-Graiwer, Christian Essrich, Katie J. Edwards, Jacqueline Pajda, Christopher J. Davis, Rami Bhadresh, Clarence R. Hurt, Beverly Freeman, Vishwanath R. Lingappa, Colm A. Kelleher, Marcela V. Karpuj

**Affiliations:** Prosetta Antiviral Inc, San Francisco, CA USA; NIAID, Ft Detrick, MD USA; DuPont Industrial Biosciences, Palo Alto, CA USA; Global Blood Therapeutics, Inc, Palo Alto, CA USA; CUBRC, Inc, Buffalo, NY USA; Life Technologies, Inc, Grand Island, NY USA; Biomedical Advanced Research & Development Authority, Office of the Assistant Secretary of Preparedness & Response, U.S. Department of Health & Human Services, Washington, DC USA; Biocon Bristol-Myers Squibb, Syngene International Ltd, Bangalore, India; Bigelow Aerospace Advanced Space Studies, Las Vegas, NV USA; BioA2Z, Inc, San Francisco, CA USA; Faculty of Medicine, Bar-Ilan University, 8 Henrietta Szold, Safed, 1311502 Israel

**Keywords:** Cell-free protein synthesis, Rift Valley fever virus, Antivirals, Capsid nucleoprotein oligomerization, Wheat germ extracts

## Abstract

**Background:**

Viral capsid assembly involves the oligomerization of the capsid nucleoprotein (NP), which is an essential step in viral replication and may represent a potential antiviral target. An in vitro transcription-translation reaction using a wheat germ (WG) extract in combination with a sandwich ELISA assay has recently been used to identify small molecules with antiviral activity against the rabies virus.

**Results:**

Here, we examined the application of this system to viruses with capsids with a different structure, such as the Rift Valley fever virus (RVFV), the etiological agent of a severe emerging infectious disease. The biochemical and immunological characterization of the in vitro*-*generated RVFV NP assembly products enabled the distinction between intermediately and highly ordered capsid structures. This distinction was used to establish a screening method for the identification of potential antiviral drugs for RVFV countermeasures.

**Conclusions:**

These results indicated that this unique analytical system, which combines nucleoprotein oligomerization with the specific immune recognition of a highly ordered capsid structure, can be extended to various viral families and used both to study the early stages of NP assembly and to assist in the identification of potential antiviral drugs in a cost-efficient manner.

**Reviewers:**

Reviewed by Jeffry Skolnick and Noah Isakov. For the full reviews please go to the Reviewers’ comments section.

**Electronic supplementary material:**

The online version of this article (doi:10.1186/s13062-016-0126-5) contains supplementary material, which is available to authorized users.

## Background

*Bunyaviridae* comprise the largest viral family with over 350 viral species in five genera [1, 2]. Many Bunyaviruses, such as Rift Valley fever virus (RVFV), are significant pathogens in humans, animals, and plants [3, 4]*.* RVFV has recently been included in the WHO list of pathogens potentially capable of causing major epidemics. Similarly, as a prototype of emerging/re-emerging pathogens, RVFV is classified as a Category A High-Priority Pathogen by the NIH and is on the CDC select agent list [5]. Currently, no licensed vaccine or therapeutics exist for use as medical countermeasures against this potentially deadly disease of humans and animals [6, 7].

The nucleoprotein (NP) capsid creates a protective environment for the viral genome; therefore, the structural integrity of the capsid is essential for viral replication and the expression of viral genes. In the past, wheat germ (WG) extract cell-free protein synthesis (CFPS) of the viral NP alone has been shown to be sufficient to recreate the assembly of spherical NPs [8]. Previous studies have shown similarity between CFPS-produced highly ordered filamentous structures (HOFS) of Hepatitis C virus (HCV) NPs and authentic viral NPs in their biochemical properties [9–11]. More recently, CFPS has been used to screen for small molecules that block NP assembly of the rabies virus (RABV), which exhibits a bullet-shaped morphology [12]. The compounds identified have been tested and shown to be active against the target viral family (*Rhabdoviridae*) in vivo.

The NPs within the *Bunyaviridae* family (which consists of multiple 27 KDa chains) form a flexible filamentous ribonucleoprotein (RNP) complex with the tripartite RNA genome of negative or ambisense polarity [13]. RVFV NPs assemble into a flexible serpentine-like structure [14], which is a stabilized multimeric form that further assembles into hexameric rings when bound to RVFV RNA [15]. Several studies have shown that NP structural integrity is essential for the survival of the RVFV [16–20].

In this report, the RVFV NP was generated by CFPS and assembled into HOFS with biochemical and biophysical characteristics identical to those of the authentic RVFV NPs. Furthermore, by modifying the CFPS conditions and by applying a combined sucrose and glycerol fractionation procedure, we were able to distinguish between HOFS and intermediately ordered assembled structures (IOAS) of the filamentous RVFV NP form. We found that the system can be used to determine the essential components for in vitro assembly and to screen small molecules and cyclic peptides for activity against RVFV NP formation, thus identifying the antiviral compounds that are active in an in vivo cell infection system. This approach has broad implications for the study of other viruses with various capsid structures.

## Results and Discussion

### RVFV NP structures produced in the CFPS are similar in size and buoyant density to authentic RVFV NP

Earlier studies of several viral families have demonstrated the assembly of NP into high-molecular-weight structures, by using WG CFPS directed by viral nucleoprotein gene templates only. These studies, which did not involve the addition of viral genomes or other structural proteins, sought to recreate a putative pathway of HCV NP spherical assembly complexes. The structures displayed a distinctive migration pattern in sucrose density gradients and exhibited a striking similarity to authentic viral HCV NP in terms of protease sensitivity, buoyant density and electron microscopy appearance [8, 10–12, 21]. However, these capsids were icosahedral or bullet-shaped.

**Fig. 1:**
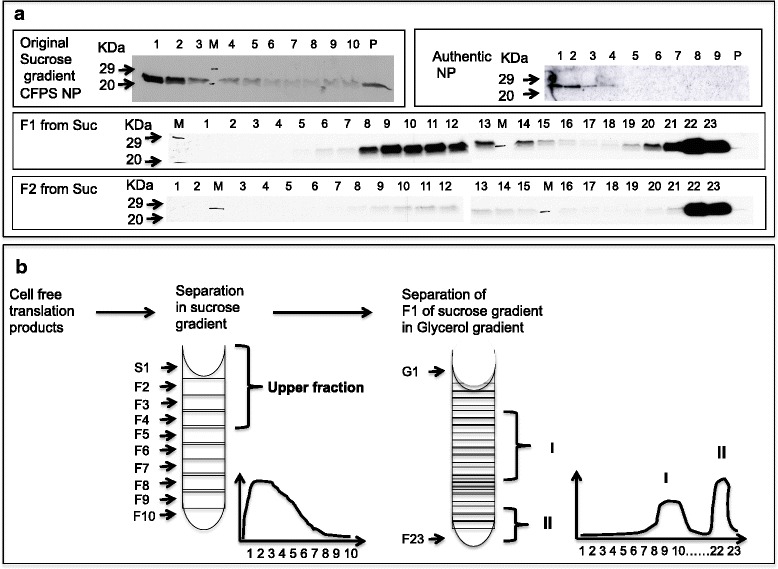
Glycerol gradient characterization of the CFPS-generated RVFV NPs, collected from different fractions of the sucrose gradients. **a** Left upper panel: SDS-PAGE analysis of the sucrose gradient fractions of radiolabeled RVFV NP translated *in vitro*. Right upper panel: western blot analysis of the authentic RVFV NP. Middle panel: SDS-PAGE analysis of glycerol gradients, on which sucrose fraction 1 was analyzed. Lower panel: SDS-PAGE analysis of glycerol gradients on which sucrose fraction 2 was analyzed. **b** Schematic representation of the two consecutive gradients used for the analysis of the *in vitro* generated RVFV NP. Note that the upper sucrose gradient fractions generated two distinct peaks on the subsequent glycerol gradient. These peaks are denoted I (middle peak) and II (bottom peak) and represent partial and higher-order assembly structures, respectively (see below)

To address whether the assembly of NP high-order, flexible filamentous structures could also be achieved in this in vitro transcription-translation system, DNA encoding RVFV NP was transcribed and then translated with a modified WG CFPS [12]. Newly synthesized RVFV NP, in the presence of ^35^S-radiolabeled methionine, was subjected to sucrose gradient sedimentation and further detected by SDS-PAGE and autoradiography (Fig. 1).

Interestingly, unlike the RABV NP, which sedimented to the bottom of the sucrose gradient [12], and the HCV NPs [8], which sedimented to the middle of the sucrose gradient, the 27 kDa newly synthesized RVFV NP was observed to sediment predominantly in the top and second fractions of a sucrose step gradient (Fig. 1a, left panel). As expected, the authentic RVFV NP comigrated in these same fractions (Fig. 1a, right panel). We hypothesized that the in vitro-generated product visualized on the sucrose gradients in both the top and middle fractions contains HOFS and IOAS, which cannot be distinguished by sucrose gradient sedimentation.

### Detection of in vitro*-*generated putative RVFV NP flexible filamentous assembly intermediates

To distinguish between RVFV NP HOFS and IOAS, the CFPS-RVFV NPs at the top running fractions of the sucrose gradients were further subjected to separation on an additional glycerol density gradient to provide a higher resolution of the different structures.

The separation of these two top sucrose gradient fractions (Fig. 1a) on a glycerol gradient indeed generated two distinct peaks (marked I and II in Fig. 1b), which were further inspected by immunoprecipitation analysis with an anti-RVFV NP monoclonal antibody (Additional file 1: figure S1). This antibody, which recognizes only the native structure of RVFV NPs, reacted only with the bottom glycerol fraction, suggesting that this fraction contains the RVFV NP HOFS and that the middle peak may represent putative intermediate steps in the assembly process.

The intermediate structures were susceptible to proteinase K digestion (Additional file 1: figure S1f and g lanes 1 and 2), whereas the higher-order filamentous structure RVFV NP that sedimented to the bottom of the glycerol gradients was relatively resistant to PK digestion (Additional file 1: figure S1f and g lane 3). The separation of these two peaks required 16 h of centrifugation (Additional file 2: figure S2b). Notably, a higher proportion of the putative intermediate structures was obtained by using a low amount of template DNA in the CFPS compared with that obtained with a high amount of template DNA (Additional file 3: figure S3). The intermediate structures were also favored when the reaction was performed at 26 °C rather than at 37 °C (Additional file 4: figure S4a and S4b, respectively). Interestingly, the glycerol gradient profile obtained with the RVFV NPs generated with the CFPS (27 kDa) was similar to those obtained with other HOFS NP viral structures from phylogenetically related viral families (HNTN and LASV) but different from those obtained with spherical viral NPs (for example HCV) (Additional file 5: figure S5).

Together, the data strongly suggest that the glycerol gradient “peak I” (see Additional file 1: figure S1) indeed represents an intermediate precursor structure of the HOFS, which reproducibly sedimented in “peak II”. To further confirm the presence of IOAS in the CFPS products, RVFV native NP structures generated in transfected HEK 293 cells were also subjected to sucrose gradient sedimentation followed by glycerol gradient sedimentation, as described above. These native RVFV NPs expressed in the HEK 293 cells sedimented at the top of the sucrose gradients, similar to the authentic RVFV NPs and the CFPS-translated RVFV NPs. However, as expected, the separation of the top sucrose fraction (Additional file 2: figure S2c, top panel) on glycerol gradients (Additional file 2: figure S2c, bottom panel) resulted only in the generation of the bottom sedimentation peak and did not reveal the putative assembly intermediates. These data also suggest that the glycerol-gradient middle peak represents intermediate-assembly structures. These intermediate structures were absent in the transfected HEK 293 cultures due to the large quantity of synthesized RVFV NP chains at 37 °C, which favored the generation of HOFS. Together, the data strongly suggest that by combining sucrose and glycerol gradients, it is possible to distinguish between HOFS and IOAS.

### CFPS RVFV NP deletion mutants that do not assemble into RVFV NP HOFS co-migrate with putative RVFV NP IOAS on glycerol gradients

It has previously been demonstrated that RVFV NP HOFS formation relies on the dimerization of RVFV NP chains, which, in turn, depends on the integrity of the NP [16]; accordingly, mutated forms of NP that exhibit deletions are affected in their ability to assemble into RVFV NP HOFS [19]. Sucrose density-gradient separations of the products encoded by RVFV NP N- or C-terminal deletion mutants were carried out in the CFPS under conditions that enabled the generation of both RVFV NP IOAS and HOFS. When the top sucrose fractions of these products were fractionated on glycerol gradients, both N- and C-terminal deletion mutants of RVFV NP preferentially sedimented to the middle fractions (Additional file 2: figure S2e and 2f, respectively), whereas the wt RVFV NP sedimented both at the middle peak, representing the intermediate structures, and at the bottom peak, suggesting the presence of RVFV NP HOFS (Additional file 2: figure S2d). These results indicate that the expression of RVFV NP mutants by CFPS mimics the natural patterns of these oligomeric structures [20].

### The lower-order assembly structures represent precursors of the higher-order assembled NPs

**Fig. 2:**
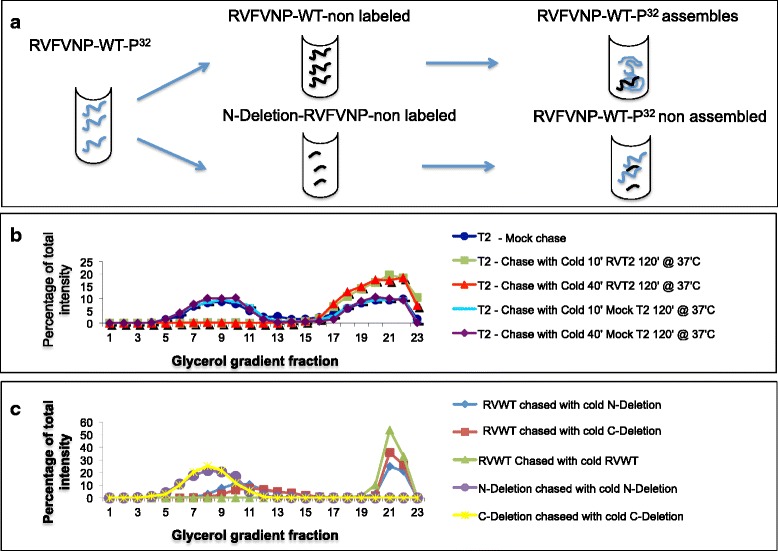
Partial and full conversion of intermediate capsid structures into highly ordered capsids by nonradioactive CFPS-products. **a** Schematic illustration of the chase experiments. **b** Glycerol-gradient fractionation profiles of the radioactive material generated by CFPS under conditions favoring intermediate-assembly structures and “chased” by addition of the intermediate-assembly structures of nonradioactive products. As indicated: dark blue, “mock” chase experiment (addition of buffer only); green, “chase” experiment (addition of nonradioactive intermediate structures). The chase was performed for 120 min at 37 °C. **c** Glycerol-gradient fractionation profiles of the radioactive material generated by CFPS “chased” by the addition of nonradioactive products involving N-terminal or C-terminal deleted NP forms. Red, reaction initiated with full-length NP forms and chased with nonradioactive full-length forms (similar to panel B); light blue, reactions initiated with full-length forms and “chased” with N-terminal deleted NP; red, reactions initiated with full-length forms and “chased” with C-terminal deleted NP; purple, reactions initiated and chased with N-terminal deleted forms; yellow, reactions initiated and chased with C-terminal deleted forms

To further demonstrate that the product sedimenting in the glycerol-gradient middle fraction represented bona fide intermediate structures, we conducted “chase” experiments, which addressed whether these S^35^-labeled RVFV NP IOAS could convert into HOFS after addition of unlabeled IOAS. To perform these experiments, we used assembly conditions that favored the synthesis of ^35^S labeled RVFV NP IOAS (26 °C for 10 min, see above). The nonradiolabeled RVFV NP products (obtained by conducting the in vitro reaction for 10 or 40 min at 26 °C) were then mixed with the radiolabeled reaction and incubated for an additional 120 min (Fig. 2) or 45 min (Additional file 6: figure S6). This reaction product was analyzed on glycerol gradients. The data indicated the transition of the radiolabeled IOAS to the RVFV NP HOFS, as demonstrated by a shift of the labeled intermediate peak toward the bottom of the glycerol gradient. This phenomenon was time-dependent because the chase of the radiolabeled RVFV NP for only 45 min (rather than 120 min) did not result in a complete transfer of the ^35^S into the RVFV NP HOFS (Additional file 6: figure S6). The transition of the intermediate fraction to the high-order assembly structures was only partially promoted by a “chase” involving either N- or C-terminal deleted versions of the RVFV NPs, thus further substantiating the conclusion that the intermediate fraction represents the precursor of the RVFV NP HOFS (Fig 2c). Notably, when the reaction was both initiated and chased with the deletion mutants, no HOFS were detected. The assembly of the intermediates into high-order NPs, as evidenced by the “chase” experiments, was independent of ATP because the inhibition of ATP by addition of Apyrase did not affect the accumulation of the assembled structures (Additional file 7: figure S7). Notably, this is not the case with other in vitro virion assembly assays treated in the exact same manner [12, 22, 23]. These observations strengthen the idea that the low-order assembly structures represent authentic intermediates, which possess the biophysical characteristics necessary for their spontaneous transition to fully assembled structures.

### Converting the CFPS reaction to a moderate-throughput plate screen

**Fig. 3:**
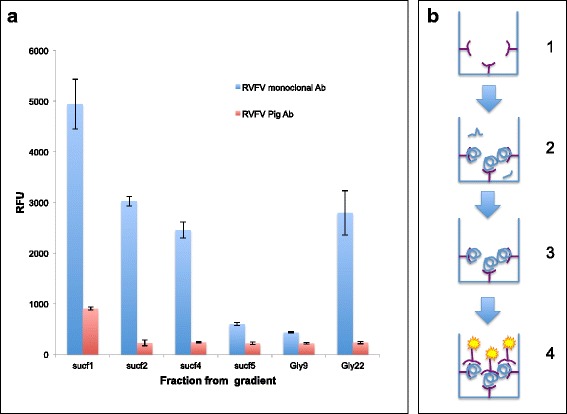
HOFS are both preferentially captured and detected in the ELISA sandwich assay. **a** RVFV NPs derived from the top of the sucrose gradient and the bottom of the glycerol gradient were preferentially detected by the anti-RVFV NP monoclonal antibody. **b** Diagram of the CFPS in combination with the plate assay screening setup. I. The plates are initially coated with first Ab. II. Equal amounts of CFPS RVFV NP are loaded onto each well. III. The plates are washed with PBS containing 1 % Triton X-100. IV. The fluorescently labeled anti-RVFV NP monoclonal antibody detect only the RVFV NP oligomers

By analogy with the successful screens for RABV with a bullet-like capsid shape [12], we adapted the assembly assay characterized above to a moderate-throughput screen that could be further scaled up into a high-throughput screening method for the identification of small molecules for blocking the RVFV NP assembly pathway. A monoclonal antibody against the authentic RVFV NP capsid was generated by USAMRIID, and polyclonal pig and rabbit antibodies against various RVFV NP peptides were generated (all available from Prosetta Biosciences). CFPS RVFV NP was translated, and an equal concentration of the labeled protein was exposed to the various antibodies. The monoclonal antibody had a higher affinity than the other antibodies in native IP conditions (Additional file 8: figure S8a). The results indicated that protein G-purified coating monoclonal antibody provided the highest signal-to-noise ratio at a dilution of 1:250 and the biotinylated detection antibody was used at a final dilution of 1:126 (Additional file 8: figure S8b). As predicted, using the monoclonal antibody against RVFV NP for ELISA capture (Fig. 3b-I) and ELISA detection (Fig. 3b-IV), resulted in superior sensitivity (a higher signal-to-noise ratio) than did use of the polyclonal porcine or rabbit anti-RVFV NP antibodies (Fig. 3a and Fig 3b-I). The synthesis was carried out in a 384-well plate, in which a common translation reaction mix was added to wells containing either 1 % DMSO or small molecules at a concentration of 25 μM dissolved in 1 % DMSO. At the end of the synthesis and assembly reactions that resulted in the assembly of putative higher-structure filamentous RVFV NP as described above, the reaction products were transferred to a second 384-well plate precoated with the monoclonal antibody recognizing only the higher-structure RVFV NP filamentous structures (see Additional file 1: figure S1d, Additional file 8: figure S8a and the scheme illustrating this process of selective detection in Fig. 3b-II and Fig. 3b-III). The HOFS of the RVFV NP were evaluated by coating the washed capture plate with the same biotinylated monoclonal antibody, which allowed the detection of a fluorescence signal when incubated with streptavidin HRP and a fluorogenic HRP substrate (Fig. 3b-IV). Small molecules that inhibited the assembly were evaluated for their inhibition of fluorescence, measured in relative fluorescence units (RFU). To avoid false positive signals, which may be due to nonspecific inhibition of protein synthesis, the mRNA for eGFP was added to the cell-free translation reaction to enable the quantification of the overall protein synthesis by fluorescence.

**Fig. 4:**
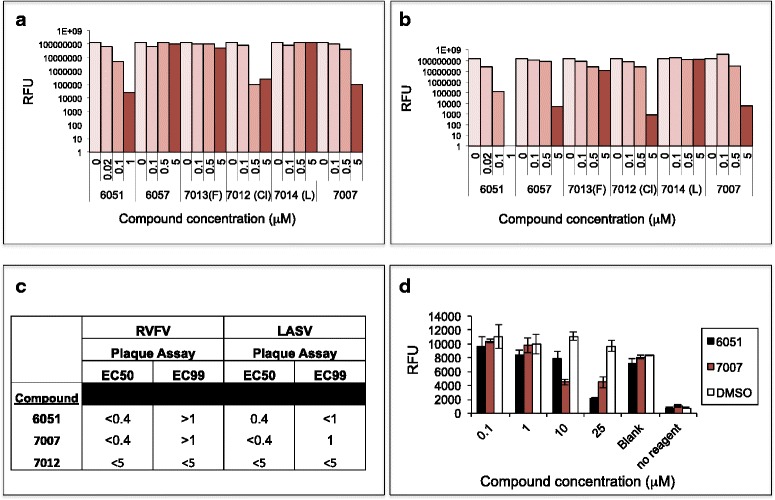
Inhibitory small molecules identified using the CFPS have an inhibitory effect in live virus assay. **a** Various compounds demonstrated a dose-dependent inhibition of RVFV NP assembly in the plate assay. **b** Similar compounds were effective against LASV NP assembly. **c** The selected “hit” compounds were potent in the live virus assay for both RVFV and LASV. **d** Exposure of the compounds to HEK-293 cells indicates no toxicity in their active concentration

**Fig. 5:**
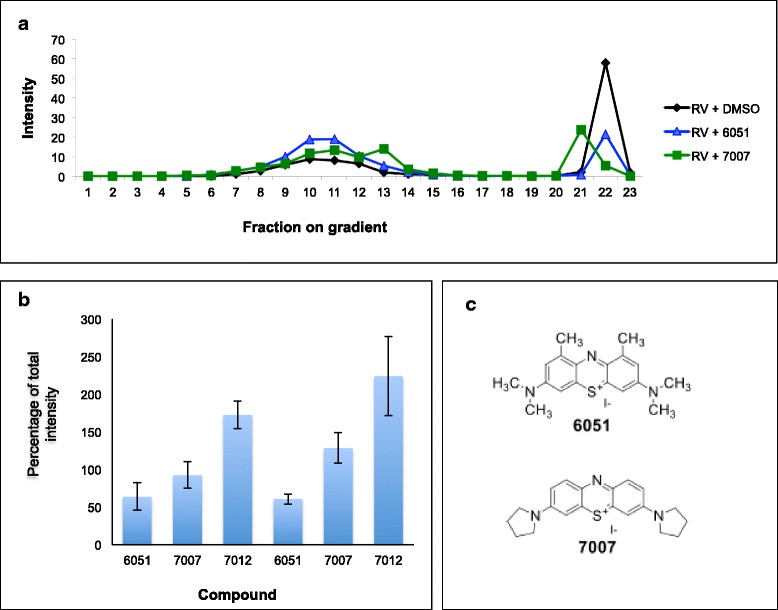
Small molecules selected by the plate assay inhibit HOFS formation. **a** The CFPS of RVFV NP was performed in the presence of compounds 6051, 7007 or 1 % DMSO and loaded onto sucrose gradients. Sucrose fraction 1 was loaded onto glycerol gradients. **b** Chemical structures of selected potent compounds

Over 10,000 compounds were screened, and a number of “hits” were identified. These compounds demonstrated inhibition of NP assembly in a dose-dependent manner (Fig. 4a) and were assessed in a yield reduction assay for activity against infectious RVFV in cell cultures (Fig. 4c). The selected compounds, which promoted the inhibition of NP assembly, were confirmed for their ability to prevent the formation of the high-order assembled structures generated by CFPS, as visualized by glycerol gradient sedimentation (Fig. 5a).

**Fig. 6:**
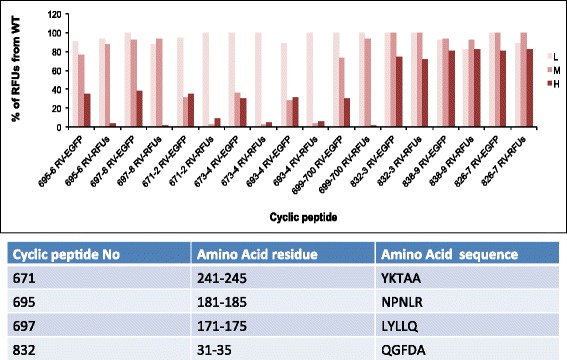
Cyclic peptides representing RVFV N- and C-terminus inhibit the assembly of IOAS into HOFS. Cyclic peptides, representing different residues within RVFV NP, at various concentrations (H, High = 20 μM; M, Medium = 2 μM; L, Low = 0.2 μM) were diluted in 1 % DMSO and incubated in the CFPS assay. To control for the effect of the cyclic peptides on protein synthesis, eGFP was cotranslated in the presence of the various compounds

The structures of compounds 6051 and 7007, which had the lowest drug concentration (0.4 μM) that inhibited 50 % of viral replication (EC50) in the yield reduction assay (Fig. 4c), are illustrated in Fig. 5c. No toxicity was observed when the HEK cells were exposed for 24 h to each of these compounds at a concentration of 1 μM (Fig. 4d). Using a similar screen of small molecules against LASV (to be discussed in greater detail elsewhere), the same compounds were selected as “hits” (Fig. 4b) and were validated as active against infectious LASV in cell cultures (Fig. 4c). Interestingly, cyclic peptides representing residues within the N- and C-terminal regions of the RVFV NP were also found to be potent inhibitors of assembly into HOFS (Fig. 6).

## Conclusions

The biochemical characterization of RVFV NP CFPS demonstrates the ability to form intermediate and higher-structure filamentous RVFV NP within the same in vitro translation reaction. Therefore, the exact conditions that favor monomers over any oligomeric filamentous structure and the methods to distinguish these two are essential to the establishment of a high-throughput screening system to identify small molecules that interfere with RVFV NP assembly or that lead to the disassembly of existing higher-structure filamentous RVFV NPs or intermediates.

Our results validate the use of CFPS in combination with sandwich ELISA assays based on specific monoclonal antibodies and provide a moderate-throughput screening method for small molecules that block the formation of RVFV NP containing structures that are similar, if not identical, to RVFV NP HOFS.

We demonstrated that the anti-NP monoclonal antibody had a higher affinity for the native NP than the denatured NP and a higher affinity for the HOFS than the IOAS. Therefore, it is not surprising that the specificity and sensitivity of the high-throughput screening assay improved when a monoclonal antibody against the same epitope was used for capture and detection, especially when the aim was to detect the oligomerization of a single NP.

The power of this assay is substantiated by the observation that some of the compounds identified as hits against RVFV NP oligomerization were validated as robustly active against infectious RVFV in cell culture. Our data also strongly suggest that this method could be extended to other members of the viral family *Bunyaviridae* and possibly to other viruses with higher-order filamentous NP structures including LASV, a member of the family *Arenaviridae*.

Notably, an established approach that has been used to assemble putative spherical HCV NP and to identify active compounds that have been validated against the corresponding authentic viruses, can also be applied with success to higher-structure filamentous viruses such as the *Bunyaviridae.* Hence, a common set of protein-protein interactions govern higher-structure filamentous assembly across the viral family. However, this study emphasizes the importance of a) identifying the different biochemical conditions that apply to HOFS and IOAS for each viral NP, b) establishing a set of antibodies that distinguish between these two and c) using the same monoclonal antibody as a capture and detection antibody for the high-throughput system.

The small molecules that were identified as potent anti-RVFV and anti-LASV in this study, together with the one that was identified as anti-RABV in a previous study using the CFPS, have a common skeleton, namely 3,7-bis(dialkylamino)phenothiazine-5-ium derivatives. These compounds have been shown to have various biomedical applications [24], and one of them, Methylene Blue, has been demonstrated to abolish West Nile virus infection in vivo, when photoinactivated [25]. Further studies on the affinity of each structure for the various viral NPs should be carried out.

Finally, the correlation between small-molecule drugs that inhibit NP assembly and their ability to inhibit virus growth in the infectious virus inhibition assay demonstrates that inhibition of NP assembly can be used as a quick, accurate, reliable, and affordable assay method to screen for potential antiviral drugs. This CFPS method is based on the synthesis of the viral nucleoprotein at different NP assembly stages. Thus, it could potentially be used to identify drugs that inhibit NP assembly formation as well as drugs that disassemble pre-existing NPs into their intermediate structures, providing an important advantage for screening therapeutics. To our knowledge, this is the first demonstration of a small molecule that inhibits assembly of higher-order flexible filamentous structures of RVFV NP.

## Methods

Materials were purchased from Sigma Chemicals Co. or Thermo-Fisher unless otherwise noted. Affinity purified antibodies to RVFV NP are available from http://www.prosetta.com.

### Cell-free transcription, translation, and chase experiments

The cell-free transcription was performed and adapted to linked translation as previously described, with the following adjustments [10, 11, 22]. The coding region of the RVFV NP was engineered behind the SP6 bacteriophage promoter and the Xenopus globin 5′ UTR. The PCR product of the RVFV NP was transcribed in vitro to generate mRNA encoding each full-length protein. The translations were carried out in the WG CFPS system supplemented with either [^35^S] methionine at 0.2 μM (ICN Biochemicals) or with all 20 nonradiolabeled amino acids, as previously indicated [11, 22, 26]. The translations were performed as previously described [21, 26] at the various temperatures and incubation times as indicated for each experiment. The WG extracts were used at 20 % of the total translation volume unless otherwise indicated. Additional components included 0.8 μM MgOAc, 80 μM KOAc, unlabeled cysteine and methionine to a final concentration of 25 μM. This was found to be optimal for RVFV NP core translation and assembly. The translation reaction mixture contained 15.6 mM ATP, 15.6 mM GTP, and 31.3 mM CTP diluted in water. A “column buffer” (4 mM HEPES at pH 7.8 with KOH, 0.8 mM KOAc, 0.8 μM MgOAc) was used as a substitute for the dilution of T2 when loaded onto the gradient or when the transcripts were diluted. A ”chase buffer “(4 mM HEPES, pH 7.6 and 0.1 mM EDTA, pH 7) was used as a mock chase material instead of the WG extract.

### Sucrose and glycerol gradients

The translation products (30 μl unless otherwise indicated) were loaded onto the sucrose gradients, in 1 % w/v Triton X-100, 50 mM, 4 mM HEPES at pH 7.6, 100 mM KOAc, and 5 mM MgOAc. The samples were adjusted to 150 μl with the column buffer. The sucrose step gradients (2 ml) were poured by layering 60 % to 10 % (from bottom to top) sucrose stock solutions containing the same Triton and salt concentration as the sample; this was followed by 1 h of diffusion at room temperature. The gradients were then cooled to 4 °C before the samples were loaded and centrifuged in a TL-100 Beckman ultracentrifuge (Beckman Coulter Inc., Carlsbad, CA) using a TLS55 swinging bucket rotor at 50,000 rpm for 55 min at 4 °C. After centrifugation, 200 μl fractions were removed from the top of the meniscus with a pipette, and the pellet was resuspended in 200 μl of 10 % sucrose stock solution. A 20 μl aliquot of each fraction was adjusted with sodium dodecyl sulfate (SDS)-containing loading buffer (10 % glycerol, 2 % SDS, 50 mM Tris at pH 6.8, 0.001 % bromophenol blue, 50 mM DTT), analyzed on a 12 or 15 % acrylamide-SDS gel prepared using Tris-glycine buffer at pH 8.8 as previously reported [12] and visualized by autoradiography. Specific bands were quantified densitometrically with ImageJ software. As a loading control, unlabeled proteins were visualized by Coomassie staining of the gels.

Then, 2-ml glycerol linear gradients, from 10 % to 30 % in column buffer, were poured using a gradient former and cooled to 4 °C. A 75 μl aliquot of fraction 1 of the previously run sucrose gradients were adjusted to 150 μl in column buffer with a final concentration of 1 % Triton X-100, loaded onto the gradient and centrifuged at 55,000 rpm for 16 h at 4 °C with the same rotor used for the sucrose gradients. After centrifugation, 100 μl fractions were removed sequentially from the top of the tube as above. The samples were centrifuged and prepared for SDS-PAGE as described above.

### Protease treatment

RVFV NP translation was performed by CFPS at 37 °C for 1 h in the presence of [^35^S] methionine at 0.2 μM, analyzed by sucrose gradients and 75 μl of fraction 1 of the sucrose gradients was analyzed by glycerol gradients. Twenty microliter aliquots were taken from the glycerol gradient fractions of interest (F8, F9, and F22) and incubated with either buffer or proteinase K at various dilutions (final concentrations of 10, 1, and 0.1 ng/ml). Protease treatment of the samples was performed at 37 °C for 2 h and terminated by the addition of PMSF and heating to 100 °C for 2 min in SDS loading buffer. Samples were the separated on a 12 % or 15 % SDS-PAGE gel. The RVFV NP was visualized by autoradiography and quantified as previously described.

### Cell culture expression and Western blot analysis of RVFV NP

An 80 % confluent monolayer of HEK 293 cells was transfected using Transfast (Promega, Madison, WI) for non-radioactive expression of RVFV NP encoding plasmid in 10 cm dishes at 37 °C with 5 % CO_2_. Cells were lysed 48 h after transfection in 300 μl of lysis buffer (The samples were adjusted to 100 μl with 5 % v/v Triton X-100, 5 M Tris at pH 8, and 0.44 g NaCl) and protein concentration was quantified by absorbance at 280 nm. Expression of RVFV NP was confirmed by analyzing 100 μg of lysates/well on SDS-PAGE followed by immune blotting with RVFV NP specific antibodies. A 2 μl of total RVFV NP CFPS sample was used as a positive control for the Western blot analysis. The primary antibody was a rabbit polyclonal antibody diluted 1:1000 and the secondary antibody was an anti-rabbit alkaline phosphate monoclonal antibody diluted 1:5000 (Sigma Chemical Co, St. Louis, MO). All antibodies were diluted in 2.5 % dry milk in TBST 0.05 %. 1 mg of total protein from the cell lysate was analyzed on a sucrose gradient and 137.5 μl of fraction 1 from the sucrose gradient was analyzed on a glycerol gradient as described above. In order to detect the RVFV NP expression in glycerol gradients by immunoblotting, blots were incubated overnight with primary and secondary antibody.

### Immunoprecipitation and purification of the antibodies

The specific fractions from the sucrose and glycerol gradients (60 μl each) were incubated with 1 μl of 1 mg/ml RVFV NP antibody, 250 μl of 1 % Triton-containing column buffer (Triton buffer), and 20 μl of either protein G- or A-linked beads (Bio-Rad) at 4 °C on a rotating wheel for 24 h. The beads were washed three times with 1 ml of 1 % Triton buffer, and this was followed by a final wash with 1 ml detergent-free salt solution (0.1 M Tris at pH 8 and 0.1 M NaCl). A 2 % SDS-containing loading buffer (with or without DTT) was added to each sample, and samples were then heated for 2 min at 100 °C and then separated on a 12 or 15 % SDS-PAGE gel. The RVFV NP was visualized by autoradiography and quantified as previously described. The polyclonal antibodies were raised against an RVFV NP peptide in rabbits, and the RVFV NP monoclonal antibodies were generated at USAMRIID. The antibody generation, purification and labeling were performed as previously described [27].

### ELISA

The plate screens were performed as previously described with the following modifications [12]: The CFPS consisted of WG extract, RVFV NP mRNA, eGFP mRNA, amino acids and an energy-regenerating system. The CFPS were performed first under conditions that did not generate the oligomerization of the NP (26 °C for 20 min) but created enough NP to oligomerize under the right conditions (shift to 37 °C for a longer incubation time). The CFPS translation products were transferred to capture plates previously coated with a monoclonal anti-RVFV NP affinity-purified antibody. The fluorescently labeled anti-RVFV NP monoclonal antibody stained with a secondary biotinylated affinity-purified antibody was added, washed, detected by NeutrAvidin HRP, washed again and incubated with fluorescein HRP substrate Quanta Blue for 1 h. The fluorescence signal was measured at 330/425 nm (excitation/emission) Reagents were obtained from Pierce Research. RVFV NP bound to the plate was then detected by the same antibody only if more than a monomer of RVFV NP was present. Drugs blocking this oligomerization inhibit the fluorescence. To exclude false positive compounds that inhibit transcription or translation, eGFP was cotranslated with RVFV NP, and the extent of eGFP fluorescence was monitored before the CFPS product material was transferred to the capture plate.

### Image analysis and quantification

Autoradiographs and immunoblots were quantified using Epson Silver Ai scanner, Adobe Photoshop, and Image J software (Image J). Integrated densities of each band were normalized against background to obtain quantitative values for graphs and charts using Microsoft Excel.

### Virus yield reduction assays

The effectiveness of the compounds was evaluated by virus yield reduction assay using Vero E-6 cells (American Type Culture Collection, Manassas, VA). The cells were maintained in Modified Eagle’s media (MEM) with 10 % fetal bovine serum (FBS), and 1X GlutaMax (Invitrogen, Carlsbad, CA). RVFV and LASV were evaluated using 90 % confluent cells in 6-well plates. Medium was removed from cells which were infected at an MOI of 0.1 in 200 μl of medium (MEM) containing 5 % FBS and no antibiotics that contained various concentration of compound. Plates were incubated 1 h at 37 °C/5 % CO_2_ with rocking every 15 min. Media containing virus was removed and plates were washed 3X with media or phosphate buffered saline (PBS; Invitrogen). After washing, media that contained various concentrations of compound was added and plates were incubated at 37 °C/5 % CO_2_. Supernatant was collected on day 1 and 2 post-infection (PI) for viral titer determination by plaque assay.

## Plaque assay

Plaque assays for RVFV and LASV used 90–100 % confluent Vero cells in 12-well plates. Samples for titration were serially diluted 10-fold and 100 μL was added to each well. Plates were incubated for 1 h at 37 °C with rocking every 15 min. A primary overlay containing 1X EBME, 5 % FBS, and 0.5 % agarose was added to each well. Plates were incubated at 37 °C/5 % CO_2_ for 3 (RVFV) or 4 (LASV) days followed by a secondary overlay, which was the same as the primary overlay with the addition of 5 % neutral red. Plaques were counted on day 4 (RVFV) or 5 (LASV) PI.

## Toxicity of compounds in vitro

The toxicity of the compounds was evaluated in 96-well HEK cells for 24 h. The same concentration of compound used for the yield reduction assay was added to the cells and the toxicity was evaluated using Promega’s CellTiter-Glo Luminescent Cell Viability Assay (Madison, WI) according to manufacturer’s recommendations. Briefly, compounds were added to the plate in triplicate and incubated at 37 °C/5 % CO_2_. Following the appropriate incubation period, media containing test compound was removed and replaced with fresh media without compound and the cell titer glo substrate. The plates were rocked for 2 min followed by 10 min incubation at room temperature to allow the signal to stabilize. Plates were read on a luminometer (SpectraMax M5, Molecular Devices, Sunnyvale, CA) at an integration time of 500 ms.

## Densitometric quantification and statistics

Quantification of radioactive material on SDS-PAGE samples exposed to X-Ray films was determined using the NIH image J software. The data represents the average and standard deviation of three independent experiments for each condition. Bonferroni’s multiple comparison test analysis, using Prism 6 software, was used for comparisons between multiple conditions. Differences between conditions were considered to be statistically significant when *p <* 0.05.

## Reviewers’ comments

### Reviewers’ report 1: Professor Jeffry Skolnick, Buffalo center of excellence in bioinformatics, USA

This paper describes a very clever in vitro system that can be used to characterize virus capsid assembly and which can do so at high throughput. Application to Rift Valley fever virus shows that the assembly system works. Moreover, it was successfully used to identify some related small molecules, Overall this is a very nice piece of work.

Overall this is a nice piece of work which has broad ramifications with tremendous potential to characterize virus capsid formation as well as assist in the identification of novel antivirals. I do have some minor recommendations. 1. Discuss how the 10,000 molecules in the screening library was selected and how many were known to have antiviral activity for other viruses. 2. What % of molecules screened had any antiviral activity? Were any novel antivirals identified? 3. Can you target the intermediately ordered structures by a more directed approach? 4. Discuss the limitations of your cell free screening system.

#### Authors’ response

1. The 10,000 compounds represent a selected portion of a larger library of approximately 90,000 drug-like compounds in the Prosetta compound collection.
2. They have not been systematically screened for other viruses at this point, but clearly have varying degrees of antiviral activity differing from viral family to viral family. Less than 0.05 % of screened compounds were scored as hits, on average, of the viruses screened. A substantial percentage (up to 75 %) of hits were active against infectious virus in mammalian cell culture, varying from viral family to viral family. A number of these were novel anti-viral small molecules. We have selected analogs of one chemotype, the phenothiazine derivatives to illustrate the points made in this paper.
3. We have not attempted a more directed approach to targeting assembly intermediates. We believe this would be possible but was beyond the scope of the present study.
4. Our cell-free screening method, has both strengths and limitations. The obvious strength is that it allows targeting of a host-directed catalytic pathway that has not been previously accessible. The limitations however are several. First, while a number of targets are clearly shared between plant and animal extracts, some may not be, and therefore will be missed. Second, the degree of divergence from target to target between plant and animal extracts is not clear. As a result it is possible that structure-activity relationship (SAR) will not be identical between the plant and animal targets, and optimization against the one may diverge from the optimum for the other. A counter to this point is that, in addition to targeting host factors, the present screen may also pick up direct anti-viral targets, although those are likely to be less potent as they lack the catalytic power of a host enzyme. Finally, as established the current screen only allows moderate throughput of compounds. As a result, thousands, but not hundreds of thousands, of compounds can be screened in a reasonable time.

### Reviewers’ report 2: Professor Noah Isakov, Ben Gurion University of the Negev, Israel

The purpose of this paper is to utilize a cell-free in vitro transcription-translation system for the synthesis of Rift Valley fever virus (RVFV) nucleoprotein capsids (NP), characterize the in vitro generated assembled products, and test whether the extent of the in vitro assembled RVFV-NP can serve as means for the screening and identification of potential anti-RVFV drugs. Optimization of the in vitro transcription-translation system and sequential separation of the translated RVFV-NP products on sucrose and glycerol gradients yielded two distinct fractions, which are suggested to represent highly ordered filamentous structures (HOFS) versus intermediately ordered assembled structures (IOAS) of the RVFV-NP. Further analyses of assembly-defective RVFV-NP mutants, as well as ‘chase’ experiments with S35-labeled RVFV-NP substantiated the assumption that fractions containing IOAS and HOFS represent intermediates versus fully assembled NP structures. Finally, using a HOFS-NP-specific mAb, the authors developed an ELISA-based throughput system for the screening of anti-RVFV drugs, thereby overcoming the requirement for a biocontainment facility. An applicability of this model for anti-Lassa virus drug screening is also demonstrated.

This is an interesting, well-executed study, which I am happy to endorse. Minor comments:

1. The authors should compare the findings in Figs 4 & 5 and discuss the reasons for the dramatic differences in the inhibitory effects of the drugs in the ELISA (>1000-fold inhibition) versus those observed following the sucrose/glycerol separation (~2-fold).
2. Page 10: Actual data of drug toxicity tested in HEK cells (Fig. 4d) is missing from Fig. 3.
3. Page 10, 3rd paragraph: The structure of the compounds is illustrated in Fig. 5c (and not Fig. 5b, as indicated).
4. Page 10, 3rd paragraph, line 4: “exposed for 24 h ‘to’ (instead of ‘with’).
5. Additional file 9: figure S9: Correct ‘Meidia’ to ‘Media’.

#### Authors’ response

1. One possibility that might explain this difference is that in the glycerol fractions contain different intermediates and the monoclonal antibody used in the ELISA recognizes only a very specific conformation that is specifically inhibited by the compounds. This is a very interesting observation by the reviewer but was beyond the scope of the present study.
2. We thank the reviewer and we added the missing figure. A new version of Fig. 4 was created and resubmitted.
3. The requested modification was made.
4. The requested modification was made.
5. A new version of Additional file 9: figure S9 was created and resubmitted.

We thank the reviewers for improving the initial manuscript by sharing their professional feedback. We would like to extend our gratitude to the editor and the unique open review process at the Biology Direct journal that facilitates such quality of review.

## Additional files

Additional file 1: figure S1. RVFV NP characterization using an immunoprecipitation assay and proteinase K digestion (PK). Upper drawing: schematic illustration of the glycerol gradient profiles, which consist of two distinct sedimentation peaks representing putative assembly intermediates (peak I) and highly ordered assembled particles (peak II). (A) SDS-PAGE analysis of the radioactively labeled CFPS products. (B) Glycerol gradient fractions of the immunoprecipitated material resulting from a mixture of peaks I and II. (C) Glycerol gradient fractions of the immunoprecipitated material resulting from peak I. (D) Glycerol gradient fractions of the immunoprecipitated material resulting from peak II. The data strongly suggest that peak II represents native-like fully assembled capsids, whereas peak I represents assembly intermediates. (E) Radioactively labeled RVFV NP was translated for 1 h at 26 °C and equal amounts of RVFV NP from the middle fractions 8 and 9 (lanes 4 and 5) and the bottom fraction 22 (lane 6) from the glycerol gradients were loaded onto an SDS-PAGE gel and stained with Coomassie brilliant blue. (F) The same concentration of radioactively labeled RVFV NP from each fraction (lanes 1 and 2 represent fractions 8 and 9 from the glycerol gradients, and lane 3 represents fraction 22) was exposed to 100 μg/ml PK or (G) 250 μg/ml PK. As a negative control to the PK reaction, DDW was added to the same final volume as the PK. As a positive control for PK activity, 4 μg of BSA was added to 20 μl of each reaction. The PK digestion assay was performed for 2 h at 37 °C. The input of the RVFV NP was normalized before PK digestion. BSA was detected using Coomassie staining (Additional file 1: figure S1 e, f and g lower panel). (PDF 3164 kb) Additional file 2: figure S2. Glycerol gradient fractions of CFPS and 293 HEK cells expressing RVFV NPs WT and mutants. (A) Radioactively labeled RVFV NRP was translated in 26 °C for an hour and run onto a sucrose gradient. First top fraction (F1) or 2nd top fraction (F2) were rerun onto a glycerol gradient. Samples were ultra-centrifuged for 16 h at 55Kg. (B) Shorter (6 h) and longer (16 h) ultracentrifugation of glycerol gradients were compared side by side. (C) HEK 293 cells were transfected with wild type RVFV NRP. Cell lysates of transfected cells were loaded onto sucrose gradient, and thereafter, top fraction of sucrose gradient was re-loaded onto glycerol gradients. All fractions of both gradients were loaded onto SDS-PAGE gels following western blot analysis using a monoclonal antibody against RVFV NRP. RVFV NRP translated by CFPS was used on each of these gels as a positive control (PC). (D) Radioactively labeled RVFV NRP was translated in 26 °C for an hour and run onto a sucrose gradient. First top fraction was rerun onto a glycerol gradient. Samples were ultra-centrifuged for 16 h at 55Kg. Glycerol gradients of Full length wild-type NP; (E) N-terminal deleted NP mutant; (F) C-terminal deleted mutant. Note the scarcity of the high order assembly capsids in the mutant gradients (fractions 20–23). (PDF 1534 kb) Additional file 3: figure S3. Transcript concentration in the in vitro translation reaction affects RVFV NP distribution on the sucrose gradients. Note that the amplitude of peak II (see Additional file 3: figure S3), representing high-order assembly structures, required higher amounts of RNA. The reactions were carried out at 26 °C, conditions that favor the accumulation of intermediates; accordingly, a substantial amount of the material was always found in peak I, representing the assembly intermediates. See also Additional file 4: figure S4 for optimization of the conditions that enable a clear distinction between the peaks representing the partially and highly ordered assembled structures. (PDF 57 kb) Additional file 4: figure S4. Glycerol gradient fractions of CFPS-generated RVFV NPs obtained by increasing the duration and temperature of the translation reaction. A) Glycerol gradient sedimentation of the reactions carried out at 26 °C for increasing durations of translation. B) Glycerol gradient sedimentation of the reactions carried out at 37 °C for increasing durations of translation. The significance of the colors of the various curves is detailed in the legends on the right. Equal amounts of the radioactive products were loaded onto the gradients in all cases, and 10 % of the transcripts were used in the translation step. (PDF 147 kb) Additional file 5: figure S5. Glycerol gradient profiles of various CFPS-generated NPs obtained by translation/transcription of different virus families. Nucleoproteins (NP) of Hepatitis C virus (HCV), RVFV, Hantaan virus (HNTN) and Lassa fever virus (LASV) were translated for 1 h at 26 °C to enable the formation of both intermediate and highly ordered assembly structures. HCV NP has a compact capsid structure, whereas RVFV, HNTN and LASV are flexible filamentous-capsid viruses. The colors of the profiles of the various viruses are indicated on the right legend. (PDF 76 kb) Additional file 6: figure S6. Full conversion of the intermediate capsid structures into highly ordered capsids by nonradioactive CFPS-products. Glycerol-gradient fractionation profiles of the radioactive material generated by CFPS under conditions favoring intermediate-assembly structures and “chased” by the addition of intermediate-assembly structures of nonradioactive products. As indicated: dark blue, “mock” chase experiment (addition of buffer only); green, “chase” experiment (addition of nonradioactive intermediate structures). The chase was performed for 45 min at 37 °C. (PDF 53 kb) Additional file 7: figure S7. Conversion of the IOAS into IHOS by nonradioactive CFPS-products is not affected by Apyrase. Glycerol-gradient fractionation profiles of the radioactive material generated in CFPs under conditions favoring intermediate-assembly structures and “chased” by the addition of nonradioactive products for 120 min and carried out in the presence or absence of the ATP inhibitor Apyrase (final concentration of 0.5 U/μl in the cell-free reaction mixture) at 37 °C. Note that in all cases, in addition to the chase at 26 °°C, the material in peak I (intermediate assembly structures) was converted to the highly ordered assembly structures (Peak II), indicating that the process of RVFV NP assembly is indeed independent of energy but is temperature dependent. (PDF 88 kb) Additional file 8: figure S8. Specificity of various antibodies against RVFV NP tested by immunoprecipitation of the radiolabeled CFPS RVFV NP. (A) In all lanes, immunoprecipitated CFPS RVFV NPs were loaded, with the exception of lane 4, which represents the total material before immunoprecipitation (positive control), and lane 11, which represents the CFPS product of the Venezuelan Equine Encephalitis Virus NP (negative control). The following antibodies were tested: Lane 1, Monoclonal anti-native RVFV (see Additional file 1: figure S1); Lanes 2, 7, 8 and 9, four different rabbit polyclonal anti-RVFV antibodies; Lanes 3, 5 and 6, three different pig polyclonal anti-RVFV antibodies. The monoclonal antibody used for the immunoprecipitation reaction analyzed in lane 1, was also used in the high-throughput system (see text). (B) Optimization of the plate assay was performed comparing the purification of the capture Ab by ammonium persulfate (A-C, G-N) with the protein G purification (D-F, O, P), in 1:250 dilution (A-D, F-N, P) or 1:500 (E, O), whereas the biotinylated detection Ab was diluted to 1:126 (A-G, K-P) or to 1:250 (H). (PDF 113 kb) Additional file 9: figure S9. Cell fractionation indicates that the compounds access the nucleus. The most potent compounds were exposed to HEK cells at a concentration of 1 μM for 24 h, after which the nucleus was separated from the cytoplasm. The concentration of these two blue compounds could be observed by the relative higher intensity in the nucleus compared to that in the cytoplasm. (PDF 3721 kb)

## Abbreviations

RVFV: rift valley fever virus
HCV: hepatitis C virus
RABV: rabies virus
LASV: lassa virus
WG: wheat germ
NP: nucleoprotein capsid
RNP: ribonucleoprotein
CFPS: cell-free protein synthesis
HOFS: highly ordered filamentous structure
IOAS: intermediately ordered assembled structures
RFU: relative fluorescence units
