# Supplementary figures and images for: Biochemical and biophysical characterization of cell-free synthesized Rift Valley fever virus nucleoprotein capsids enables in vitro screening to identify novel antivirals
Source: Biol Direct. 2016 May 14;11:25. doi: 10.1186/s13062-016-0126-5 (PMC4867995; doi:10.1186/s13062-016-0126-5)

# Supplementary figure 1

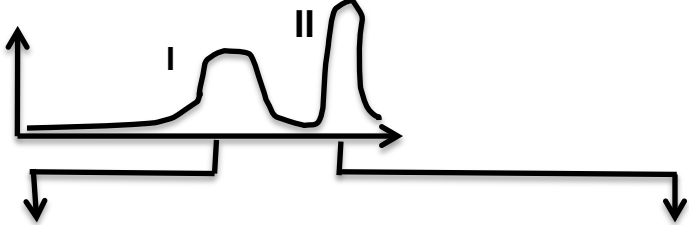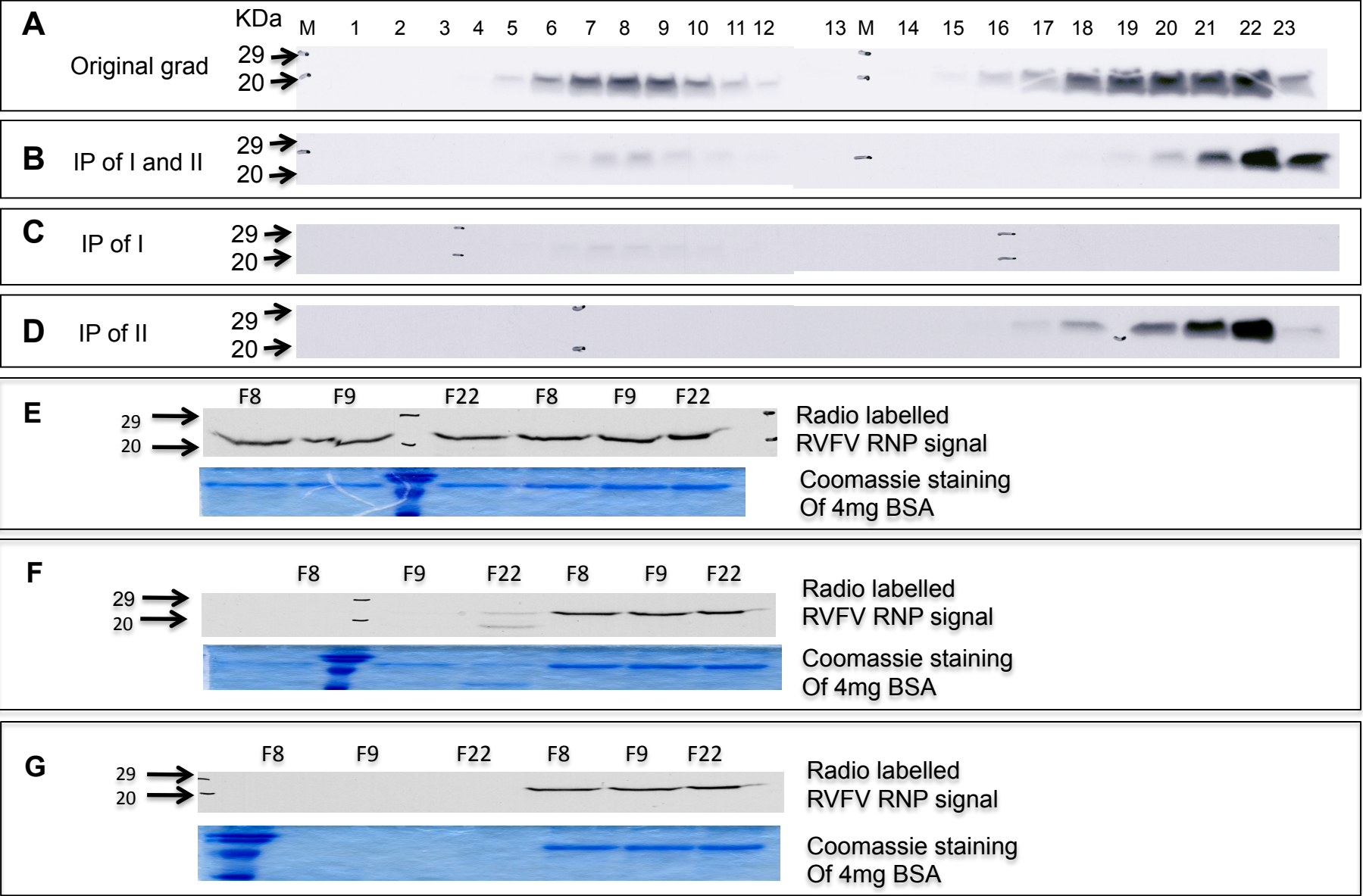

Supplement: Additional file 1: figure S1. — RVFV NP characterization using an immunoprecipitation assay and proteinase K digestion (PK). Upper drawing: schematic illustration of the glycerol gradient profiles, which consist of two distinct sedimentation peaks representing putative assembly intermediates (peak I) and highly ordered assembled particles (peak II). (A) SDS-PAGE analysis of the radioactively labeled CFPS products. (B) Glycerol gradient fractions of the immunoprecipitated material resulting from a mixture of peaks I and II. (C) Glycerol gradient fractions of the immunoprecipitated material resulting from peak I. (D) Glycerol gradient fractions of the immunoprecipitated material resulting from peak II. The data strongly suggest that peak II represents native-like fully assembled capsids, whereas peak I represents assembly intermediates. (E) Radioactively labeled RVFV NP was translated for 1 h at 26 °C and equal amounts of RVFV NP from the middle fractions 8 and 9 (lanes 4 and 5) and the bottom fraction 22 (lane 6) from the glycerol gradients were loaded onto an SDS-PAGE gel and stained with Coomassie brilliant blue. (F) The same concentration of radioactively labeled RVFV NP from each fraction (lanes 1 and 2 represent fractions 8 and 9 from the glycerol gradients, and lane 3 represents fraction 22) was exposed to 100 μg/ml PK or (G) 250 μg/ml PK. As a negative control to the PK reaction, DDW was added to the same final volume as the PK. As a positive control for PK activity, 4 μg of BSA was added to 20 μl of each reaction. The PK digestion assay was performed for 2 h at 37 °C. The input of the RVFV NP was normalized before PK digestion. BSA was detected using Coomassie staining (Additional file 1: figure S1 e, f and g lower panel). (PDF 3164 kb) [file 13062_2016_126_MOESM1_ESM.pdf]

# Supplementary figure 2

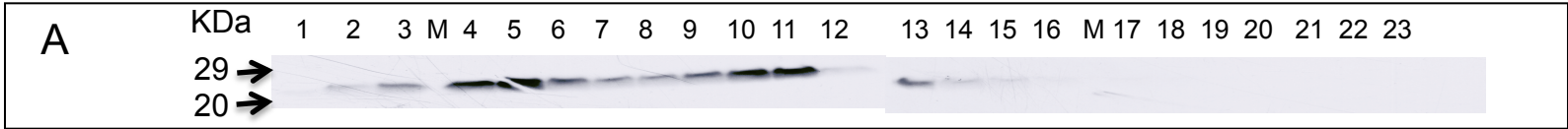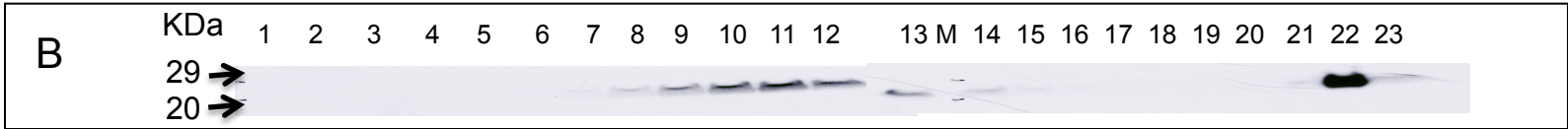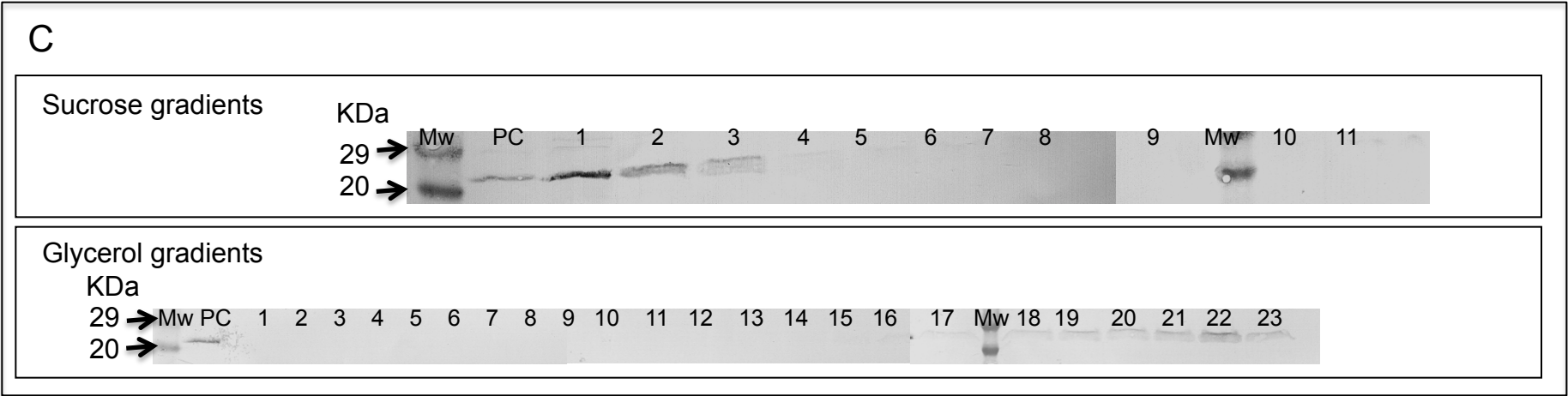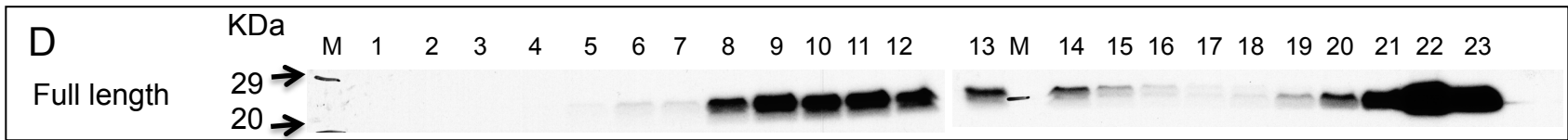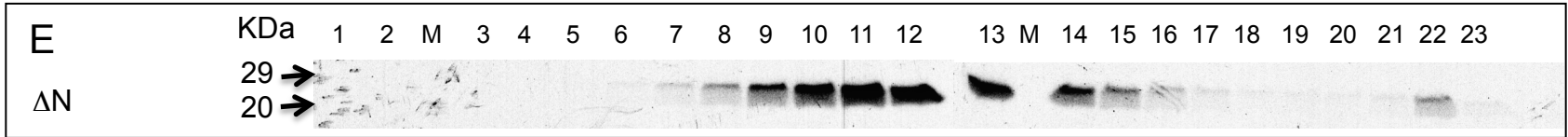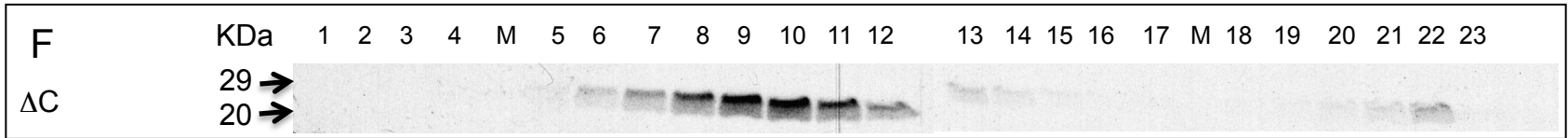

Supplement: Additional file 2: figure S2. — Glycerol gradient fractions of CFPS and 293 HEK cells expressing RVFV NPs WT and mutants. (A) Radioactively labeled RVFV NRP was translated in 26 °C for an hour and run onto a sucrose gradient. First top fraction (F1) or 2nd top fraction (F2) were rerun onto a glycerol gradient. Samples were ultra-centrifuged for 16 h at 55Kg. (B) Shorter (6 h) and longer (16 h) ultracentrifugation of glycerol gradients were compared side by side. (C) HEK 293 cells were transfected with wild type RVFV NRP. Cell lysates of transfected cells were loaded onto sucrose gradient, and thereafter, top fraction of sucrose gradient was re-loaded onto glycerol gradients. All fractions of both gradients were loaded onto SDS-PAGE gels following western blot analysis using a monoclonal antibody against RVFV NRP. RVFV NRP translated by CFPS was used on each of these gels as a positive control (PC). (D) Radioactively labeled RVFV NRP was translated in 26 °C for an hour and run onto a sucrose gradient. First top fraction was rerun onto a glycerol gradient. Samples were ultra-centrifuged for 16 h at 55Kg. Glycerol gradients of Full length wild-type NP; (E) N-terminal deleted NP mutant; (F) C-terminal deleted mutant. Note the scarcity of the high order assembly capsids in the mutant gradients (fractions 20–23). (PDF 1534 kb) [file 13062_2016_126_MOESM2_ESM.pdf]

# Supplementary figure 3

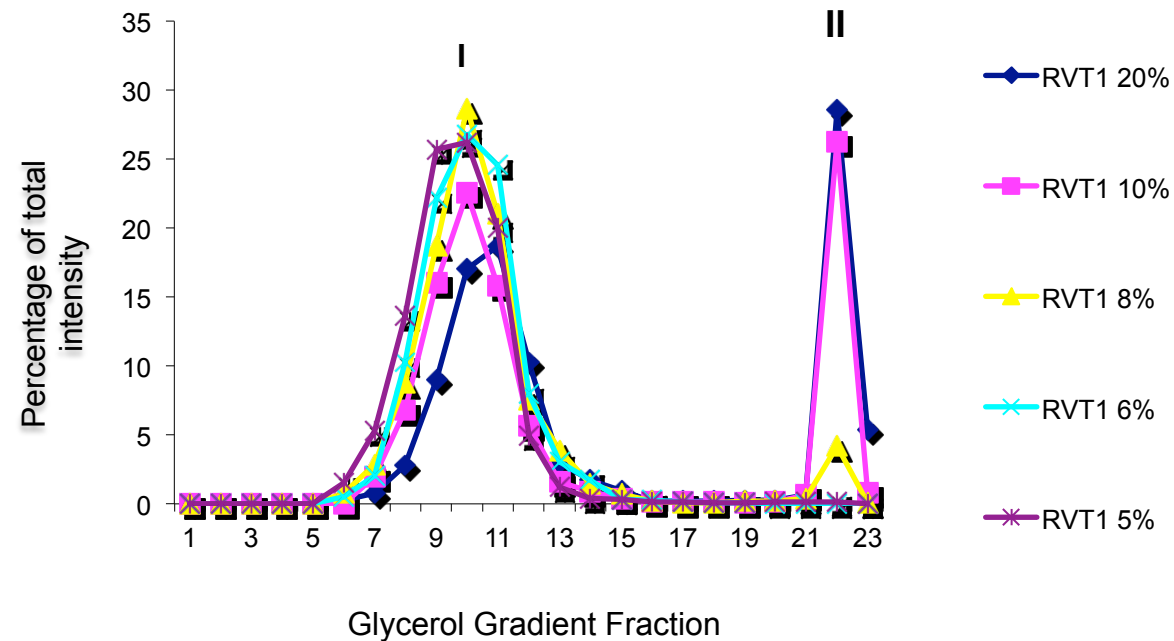

Supplement: Additional file 3: figure S3. — Transcript concentration in the in vitro translation reaction affects RVFV NP distribution on the sucrose gradients. Note that the amplitude of peak II (see Additional file 3: figure S3), representing high-order assembly structures, required higher amounts of RNA. The reactions were carried out at 26 °C, conditions that favor the accumulation of intermediates; accordingly, a substantial amount of the material was always found in peak I, representing the assembly intermediates. See also Additional file 4: figure S4 for optimization of the conditions that enable a clear distinction between the peaks representing the partially and highly ordered assembled structures. (PDF 57 kb) [file 13062_2016_126_MOESM3_ESM.pdf]

Supplementary figure 4

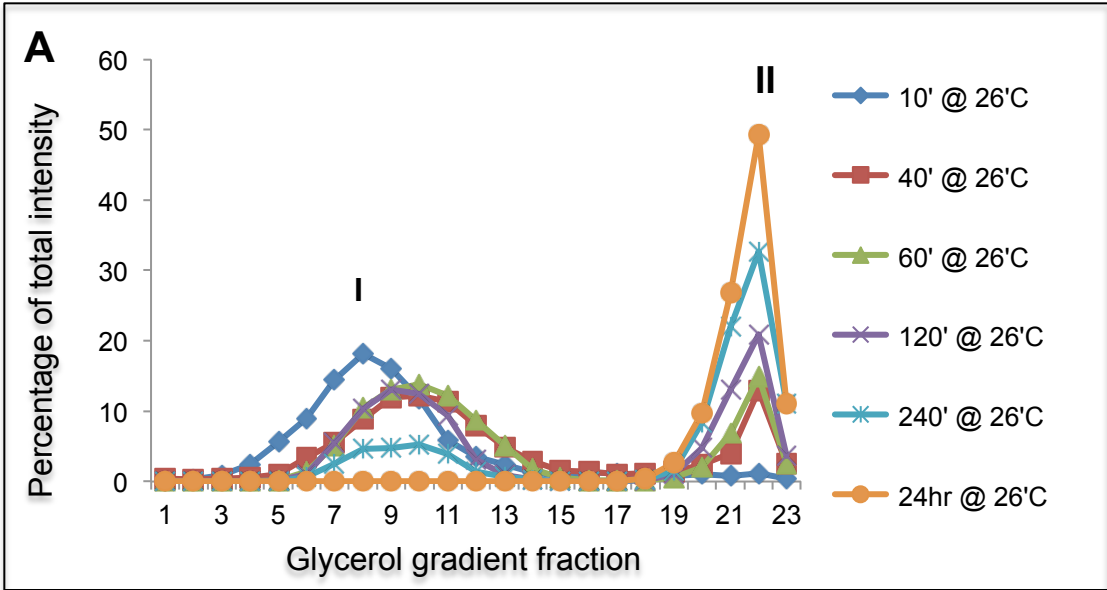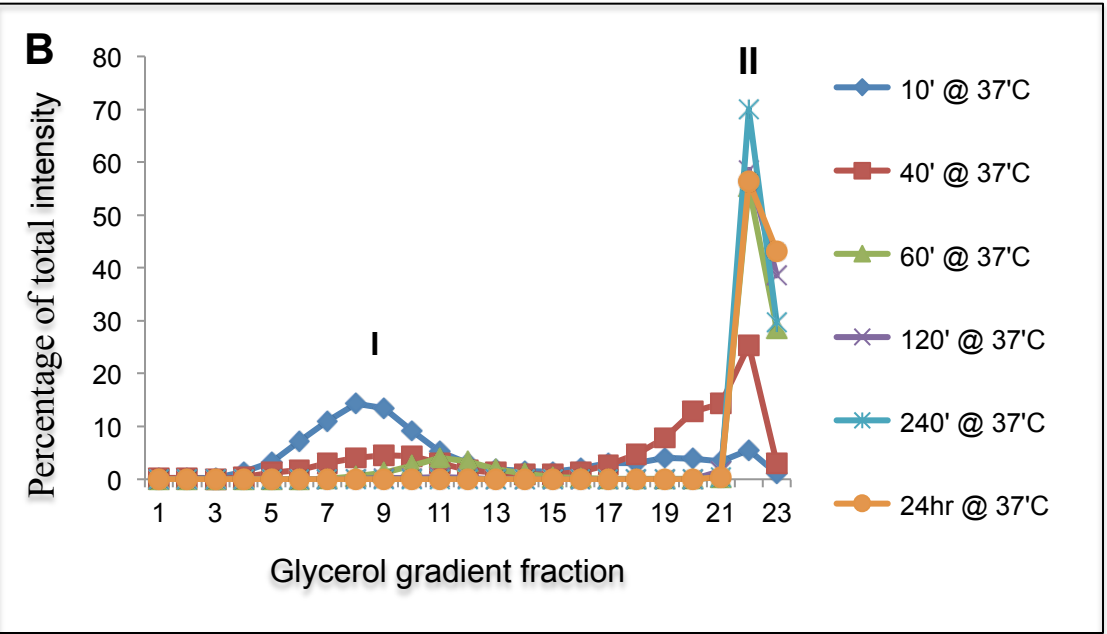

Supplement: Additional file 4: figure S4. — Glycerol gradient fractions of CFPS-generated RVFV NPs obtained by increasing the duration and temperature of the translation reaction. A) Glycerol gradient sedimentation of the reactions carried out at 26 °C for increasing durations of translation. B) Glycerol gradient sedimentation of the reactions carried out at 37 °C for increasing durations of translation. The significance of the colors of the various curves is detailed in the legends on the right. Equal amounts of the radioactive products were loaded onto the gradients in all cases, and 10 % of the transcripts were used in the translation step. (PDF 147 kb) [file 13062_2016_126_MOESM4_ESM.pdf]

Supplementary figure 5

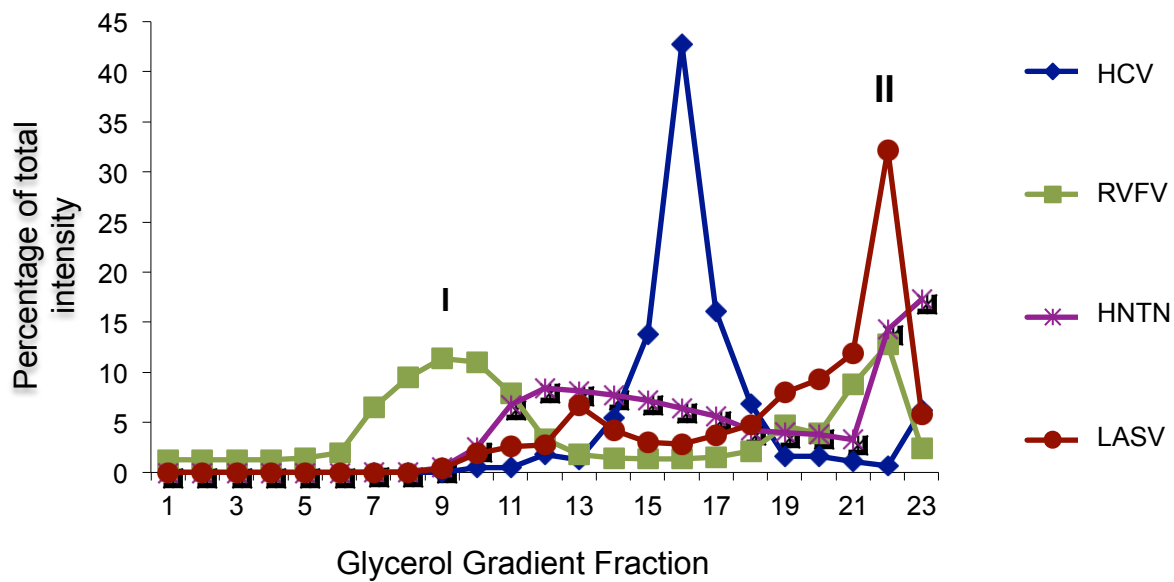

Supplement: Additional file 5: figure S5. — Glycerol gradient profiles of various CFPS-generated NPs obtained by translation/transcription of different virus families. Nucleoproteins (NP) of Hepatitis C virus (HCV), RVFV, Hantaan virus (HNTN) and Lassa fever virus (LASV) were translated for 1 h at 26 °C to enable the formation of both intermediate and highly ordered assembly structures. HCV NP has a compact capsid structure, whereas RVFV, HNTN and LASV are flexible filamentous-capsid viruses. The colors of the profiles of the various viruses are indicated on the right legend. (PDF 76 kb) [file 13062_2016_126_MOESM5_ESM.pdf]

# Supplementary figure 6

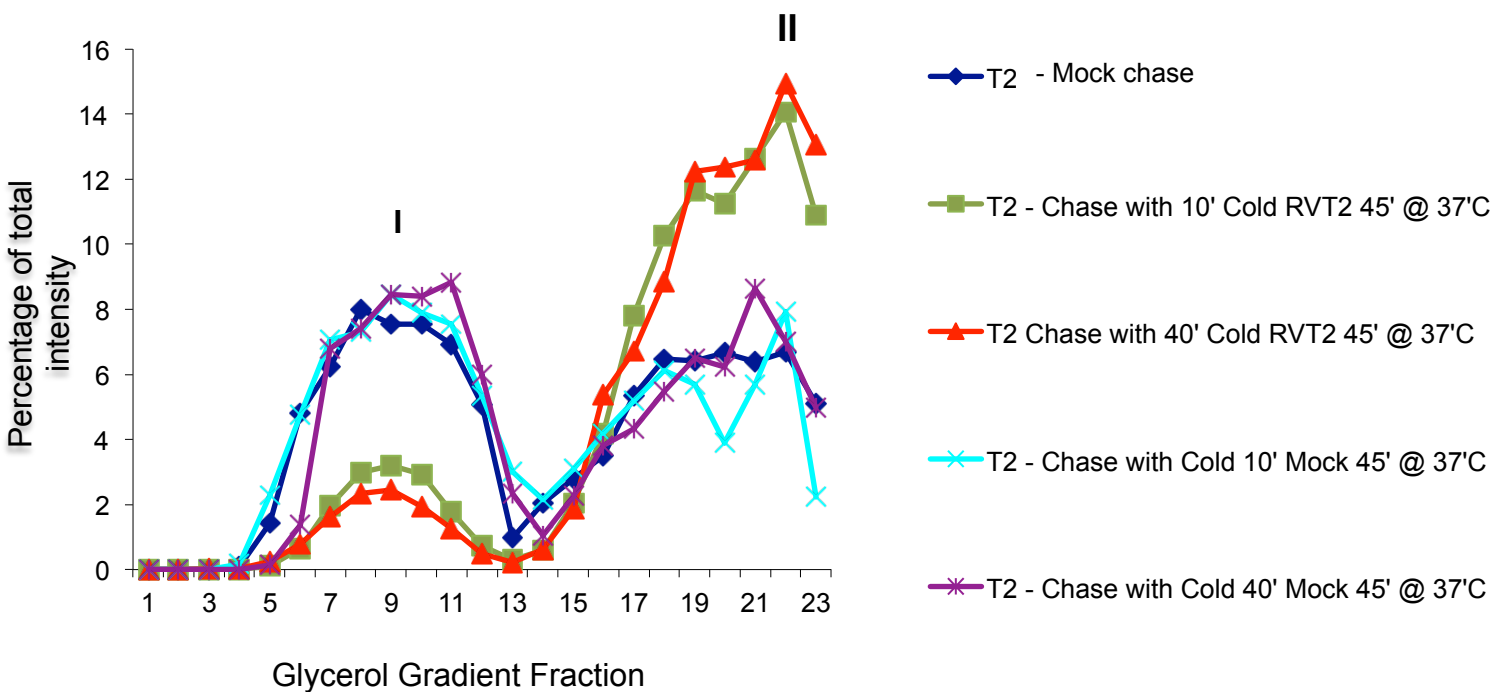

Supplement: Additional file 6: figure S6. — Full conversion of the intermediate capsid structures into highly ordered capsids by nonradioactive CFPS-products. Glycerol-gradient fractionation profiles of the radioactive material generated by CFPS under conditions favoring intermediate-assembly structures and “chased” by the addition of intermediate-assembly structures of nonradioactive products. As indicated: dark blue, “mock” chase experiment (addition of buffer only); green, “chase” experiment (addition of nonradioactive intermediate structures). The chase was performed for 45 min at 37 °C. (PDF 53 kb) [file 13062_2016_126_MOESM6_ESM.pdf]

# Supplementary figure 7

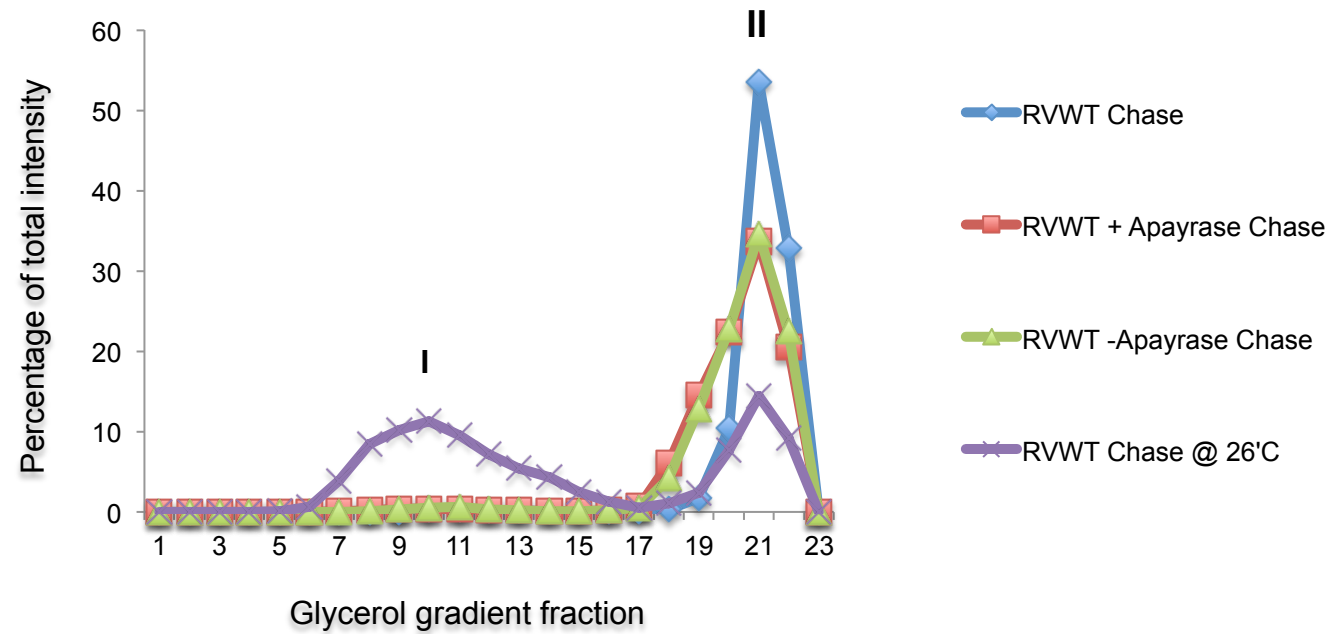

Supplement: Additional file 7: figure S7. — Conversion of the IOAS into IHOS by nonradioactive CFPS-products is not affected by Apyrase. Glycerol-gradient fractionation profiles of the radioactive material generated in CFPs under conditions favoring intermediate-assembly structures and “chased” by the addition of nonradioactive products for 120 min and carried out in the presence or absence of the ATP inhibitor Apyrase (final concentration of 0.5 U/μl in the cell-free reaction mixture) at 37 °C. Note that in all cases, in addition to the chase at 26 °°C, the material in peak I (intermediate assembly structures) was converted to the highly ordered assembly structures (Peak II), indicating that the process of RVFV NP assembly is indeed independent of energy but is temperature dependent. (PDF 88 kb) [file 13062_2016_126_MOESM7_ESM.pdf]

# Supplementary figure 8

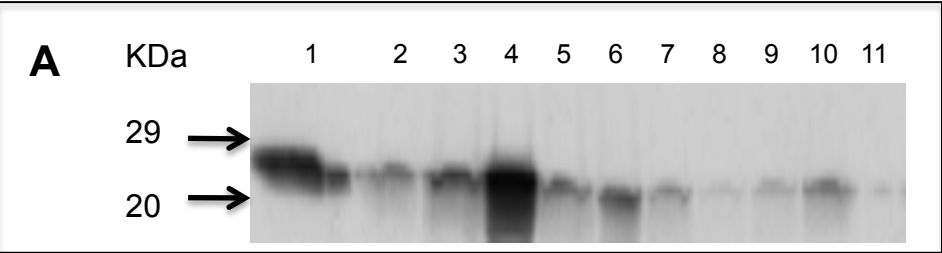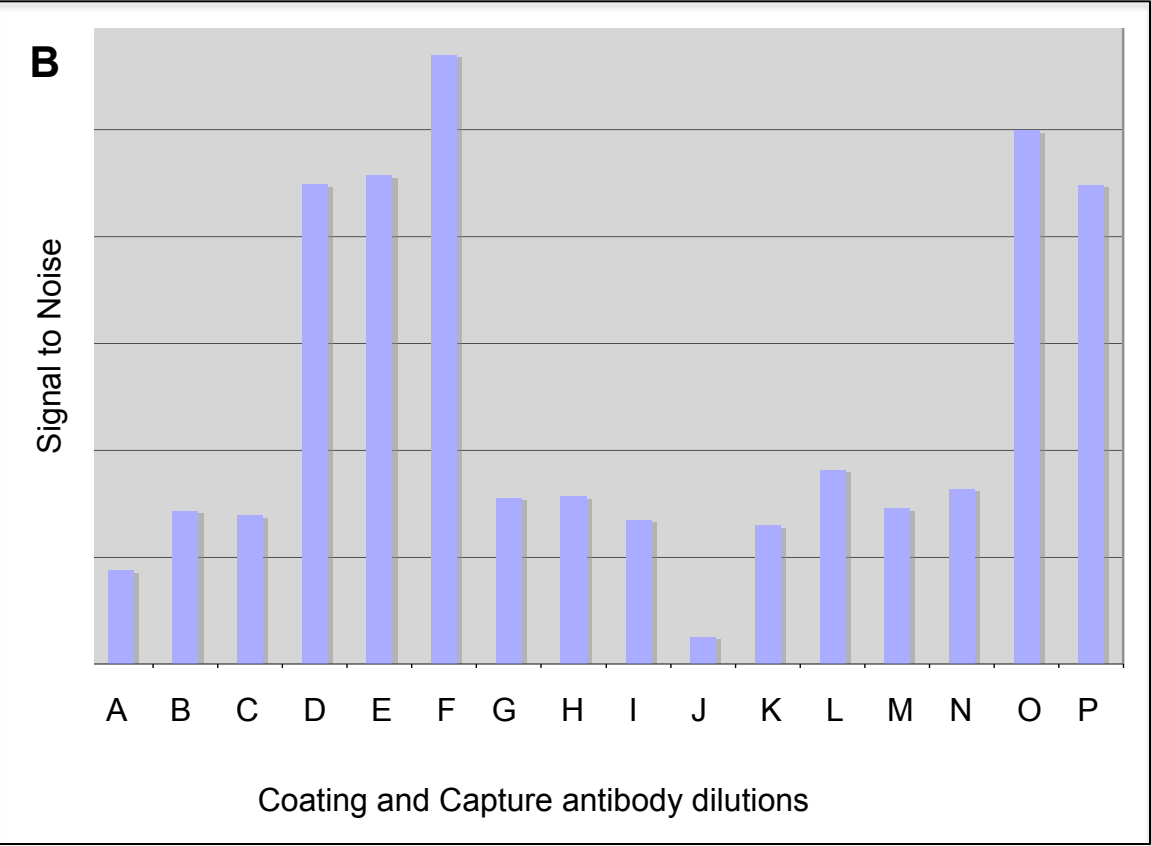

Supplement: Additional file 8: figure S8. — Specificity of various antibodies against RVFV NP tested by immunoprecipitation of the radiolabeled CFPS RVFV NP. (A) In all lanes, immunoprecipitated CFPS RVFV NPs were loaded, with the exception of lane 4, which represents the total material before immunoprecipitation (positive control), and lane 11, which represents the CFPS product of the Venezuelan Equine Encephalitis Virus NP (negative control). The following antibodies were tested: Lane 1, Monoclonal anti-native RVFV (see Additional file 1: figure S1); Lanes 2, 7, 8 and 9, four different rabbit polyclonal anti-RVFV antibodies; Lanes 3, 5 and 6, three different pig polyclonal anti-RVFV antibodies. The monoclonal antibody used for the immunoprecipitation reaction analyzed in lane 1, was also used in the high-throughput system (see text). (B) Optimization of the plate assay was performed comparing the purification of the capture Ab by ammonium persulfate (A-C, G-N) with the protein G purification (D-F, O, P), in 1:250 dilution (A-D, F-N, P) or 1:500 (E, O), whereas the biotinylated detection Ab was diluted to 1:126 (A-G, K-P) or to 1:250 (H). (PDF 113 kb) [file 13062_2016_126_MOESM8_ESM.pdf]
