# Supplementary material for: Biochemical and biophysical characterization of cell-free synthesized Rift Valley fever virus nucleoprotein capsids enables in vitro screening to identify novel antivirals
Source: Biol Direct. 2016 May 14;11:25. doi: 10.1186/s13062-016-0126-5 (PMC4867995; doi:10.1186/s13062-016-0126-5)
Supplement: Additional file 9: figure S9. — Cell fractionation indicates that the compounds access the nucleus. The most potent compounds were exposed to HEK cells at a concentration of 1 μM for 24 h, after which the nucleus was separated from the cytoplasm. The concentration of these two blue compounds could be observed by the relative higher intensity in the nucleus compared to that in the cytoplasm. (PDF 3721 kb) [file 13062_2016_126_MOESM9_ESM.pdf]

Supplementary figure 9

TX of cells 24h →

1μM compound for 24h

(resuspend cells, pool of 4X10 cm confluent plates, with media) →

Spin cells 1000g 10min

(separate sup from cells)

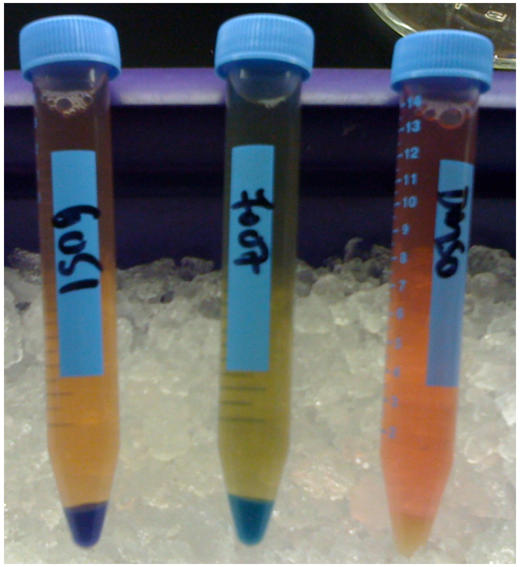

Lysis of cells in the pellet using 400μl lysis buffer

Spin cells 14,000g 30min

(separate cytoplasm from Nucleus)

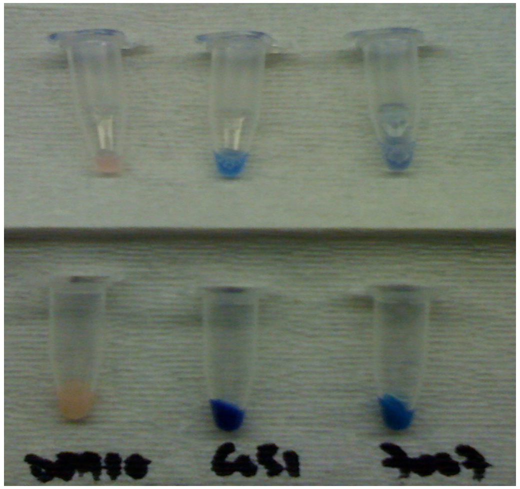

Cytoplasm

nucleus
